# Costs incurred by outpatients at a university hospital in northwestern Ethiopia: a cross-sectional study

**DOI:** 10.1186/s12913-018-3628-2

**Published:** 2018-11-08

**Authors:** Fitsum Sebsibe Teni, Begashaw Melaku Gebresillassie, Eshetie Melese Birru, Sewunet Admasu Belachew, Yonas Getaye Tefera, Befikadu Legesse Wubishet, Bethelhem Hailu Tekleyes, Bilal Tessema Yimer

**Affiliations:** 1 Department of Pharmaceutics and Social Pharmacy, School of Pharmacy, College of Health Sciences, Addis Ababa University, Addis Ababa, Ethiopia; 2 Department of Clinical Pharmacy, School of Pharmacy, College of Medicine and Health Sciences, University of Gondar, Gondar, Ethiopia; 3 Department of Pharmacology, School of Pharmacy, College of Medicine and Health Sciences, University of Gondar, Gondar, Ethiopia; 4 Research Center for Generational Health and Ageing, Faculty of Health and Medicine, University of Newcastle, Callaghan, NSW Australia; 5 Department of Pharmaceutics, School of Pharmacy, College of Medicine and Health Sciences, University of Gondar, Gondar, Ethiopia

**Keywords:** Direct medical cost, Direct non medical cost, Gondar, Indirect cost, Outpatients

## Abstract

**Background:**

Out-of-pocket expenditure constitutes high proportion of healthcare spending in low-income countries. It can affect patients’ adherence to treatments leading to serious health consequences. The objective of this study was to document costs incurred by patients visiting Gondar University Referral Hospital, in Gondar, northwestern Ethiopia.

**Methods:**

An institution-based cross-sectional study was conducted among 346 outpatients at the hospital from 2nd to 20th of May 2016. Data collection took place through interviews with patients coming to the outpatient pharmacy after finishing their visits at the different departments in the hospital. Data were collected on socio-demographic information, cost incurred before and during hospital visit as well as ownership of household items.

**Results:**

Among the 342 interviews included in the final analysis, a median total cost of 22.25 USD was incurred by patients. This constituted spending on solutions tried before hospital visit, direct medical, nonmedical and indirect costs. Among these, direct nonmedical and indirect costs constituted a large share. Medicine, transportation and waiting time during visit were major components of direct medical, nonmedical and indirect costs respectively. Total median cost was found to be predicted by residence, marital status and payment scheme used to pay for hospital services.

**Conclusions:**

Outpatients visiting the hospital incurred significant costs for illnesses/conditions associated with their visit to the hospital, the main components being nonmedical and indirect costs. Residence, marital status and payment scheme, predicted median total cost. Direct nonmedical costs and indirect costs were found to be significant components associated to the spending and loss of earning by patients and their families in their trip to and from the hospital.

## Background

According to the World Health Organization (WHO), out-of-pocket (OOP) payment is described to constitute payments made directly to health care providers when receiving service [1]. OOP spending is high in most low-income countries, representing more than half of total health expenditure (THE) in 47 low-income countries. Governments cover the remaining expense largely. In contrast, OOP payments made by individuals in the richest countries are low [2].

High OOP expenditure could lead to discontinuation of treatment or prevent from seeking health services among others due to inability to cover these costs [2]. OOP costs incurred by patients usually are the most prominent determinants of therapeutic adherence and of the effectiveness of prescribed pharmacotherapy. Even small increases in these costs can lead to potentially important reductions in medication adherence, which in turn can have serious consequences for patients’ health [3].

The high OOP could even reach to the extent of encroaching on spending for basic needs including food, clothing, housing, and others. According to WHO, about 150 million people face financial catastrophe, which involves paying over 40% of their income on health care expenditure after fulfilling their basic needs. Of these people, 100 million are pushed below the poverty line [2].

Globally, numerous studies have been conducted to estimate the level of cost incurred by patients for various health services [4–7]. A study on health services utilization and OOP expenditure at public and private health facilities of low-income countries showed that expenditure on medicine accounts for the largest share in both facilities. On average, medicines represented over 57% of outpatient OOP at public facilities and over 45% of outpatient OOP at private facilities [8]. Studies on indirect cost impacts of diseases have also been reported in terms of loss of productivity [9, 10].

In Ethiopia, poor health care financing remains a major challenge for the health system. It leaves households vulnerable to impoverishment from catastrophic health expenditures and slows progress towards health improvements by limiting access to essential health services among the poor. The situation is associated with the low proportion of government spending in the health sector, high dependence on OOP expenditure, inefficiency and inequitable resource use, and lack of harmonized predictable funding from donors [11].

Despite this, the national health expenditure in Ethiopia has been growing steadily. In 2010/11, it reached 26.5 billion Ethiopian Birr (ETB) (1.6 billion USD), up from 11.1 billion ETB (1.2 billion USD) in 2007/08. The major source of this increment in spending was funding by donors and international nongovernmental organizations. However, government spending on health increased substantially (67%) from 2.5 to 4.1 billion ETB, and household spending more than doubled when measured in ETB. The share of gross domestic product going to health reached 5.2%, up from 4.5% in 2007/08 [12].

Per capita health expenditure rose from 150.48 ETB (16.10 USD) in 2007/08 to 334.81 ETB (20.77 USD) in 2010/11 [10]. Nevertheless, it is far from the 34 USD recommended by WHO in 2001 to deliver essential health care in low-income countries like Ethiopia. It was also less than the average spent (22 USD in 2006) by 49 low-income countries with per capita income of 935 USD or less [13, 14]. According to a World Bank statistic, OOP spending in Ethiopia accounted for 32.26% of THE and 78.14% of private health expenditure in 2014. These figures are much higher than the global estimate of 18.17% of THE and 45.53% of private health expenditure [15].

Studies on the economic aspects of healthcare services in Ethiopia are generally scarce. Those concerning spending borne by patients are also few which focused on specific diseases. Among these are studies on costs related to multi-drug resistant tuberculosis [16], cervical cancer [17], reproductive health [18], maternal health [19], malaria [20], HIV/AIDS [21] and diarrhea as well as pneumonia [22].

This study aimed at documenting the level of costs incurred by outpatients visiting a university hospital in Gondar town, northwestern Ethiopia.

## Methods

### Study setting and design

The study was conducted at Gondar University Referral Hospital (GURH) in Gondar town, northwestern Ethiopia. It is a referral teaching hospital, with a catchment population of 5 million. The hospital provides inpatient and outpatient medical services in several departments. The hospital provides fee waiver for maternal care related services. The hospital has a more than 1000-bed capacity and provides service to over 200,000 patients annually [23]. The hospital serves as a referral center for four district hospitals in the area [24]. The services provided to patients referred from other institutions include chronic illnesses such as hypertension and diabetes, surgery, psychiatric care, obstetrics and gynecology among other. The study was done from 2nd to 20th of May 2016 at the outpatient pharmacy of the hospital.

An institution-based cross-sectional study design was employed in the study among outpatients treated in the hospital. The study population included adult outpatients who received service at the hospital and went to the outpatient pharmacy to pick up their prescribed medicines.

### Sampling

The sample size was determined using a single population mean formula by making a reasonable estimate of the minimum and maximum cost incurred by outpatients in the hospital to calculate the standard deviation (SD) used in the formula (one-fourth of the range). This approach has been followed as no similar studies in Ethiopia, assessing general outpatient visits, reporting spending by outpatients with information on SD were found [25, 26].

Based on the above approach, the cost incurred by outpatients is assumed to range from as low as just above 1 United States Dollar (USD) (25 ETB) to more than 22 USD (500 ETB). The SD was calculated to be 118.75 ETB. The following formula has been used; $\left[N=\frac{{\left({z}_{1-\propto}\right)}^2\times \upsigma 2}{\delta^2}\right]$, where: *z*_1 − ∝_ from the standard normal distribution was set as 1.96 at 95% confidence interval (CI). Standard deviation (σ) was estimated to be 5.44 USD (118.75 ETB) and the margin of error (*δ*) was set at 5% (0.6 USD (13.12 ETB)). The sample size was calculated to be 314.5. After adding a 10% contingency, the total sample size became 346.

In recruiting the participants, the total sample was divided equally for 15 days and each day interviews were conducted by approaching every fifth patient at the outpatient pharmacy. This assumed an average of 120 prescription filling encounters per day in the pharmacy.

### Data collection and management

A structured interviewer-administered data collection instrument was developed and checked for its face and content validity for use in the study. It was first prepared in English and translated into Amharic, official language of the country and widely spoken in the study area. It was then back translated into English, to make sure the original meaning was retained. The instrument had four parts including socio-demographic profile, the cost incurred before visit to the hospital, monetary cost and time loss associated with a hospital visit as well as questions on household items ownership (for wealth index classification). The pretest was conducted using 30 interview encounters, which were not part of the final analysis, prior to the commencement of data collection. On the basis of this, relevant modifications were instituted.

A structured face-to-face interview was conducted from 2nd to 20th of May 2016 by four pharmacy students, with patients visiting the outpatient pharmacy of the hospital after completing preceding diagnostic and treatment procedures. The data collectors were provided with a one-day training on the details of the data to be collected and interaction with study participants among others. The data was collected at the pharmacy because it is usually the last point of contact for outpatients who went through the diagnosis and other related medical procedures before leaving the hospital. Hence, the outpatient pharmacy provides the best opportunity to interview patients about their overall costs associated to their visit as the last point of contact in the hospital.

This study focused on identifying the cost incurred by outpatients and their families. Hence, it followed a patient perspective which prompted the assessment of individual/family spending for medical and non-medical expenses and indirect costs they incur. Spending included medical costs like consultation fees, investigation fees and spending on medicine(s). Nonmedical costs like transportation costs, meal related costs, lodging costs (if applicable) and other relevant spending were also assessed. Patients were asked as to how much they incurred in these specific cost components. Indirect costs considered included time loss before hospital, during travel to and from hospital as well as stay during hospital visit. In the cases where patients were accompanied by caregivers, costs associated with them were recorded. In handling costs associated to caregivers, in the cases where patients came to the hospital without caregivers, costs associated to caregivers were taken to be zero. Hence, calculations for caregiver related costs were made with total sample size in consideration.

The analytical horizon/timeframe considered in this study was the last visit patients made to the hospital associated with their medical condition(s) during the study period.

### Data entry, analysis and interpretation

The data collected was entered to, cleaned and analyzed using Statistical Packages for Social Sciences (version 23) [27]. Descriptive statistics involving frequency and median was used and findings were presented in tables and a graph. The total cost incurred by each patient/family was calculated by adding the amount spent on solutions tried before visiting the hospital (if any), the amount of money spent for direct medical service and nonmedical items and services as well as indirect costs. The cost data were collected in ETB and was converted to USD in 2016 rate, in the analysis and reporting of the findings (1 ETB = 0.0458 USD) [28]. Indirect cost was calculated by multiplying total time lost in days, both patient and caregiver-related, by the daily earnings of patients and caregivers.

The distribution of the data on total cost incurred by patients and their families was highly skewed to the right. Due to this, descriptive analyses and presentation of the data involved use of median instead of mean as the former is less prone to influence by outliers. Analysis of the association of various socio-demographic variables with total cost also took the skewed distribution in to consideration. Hence, a quantile regression on the median total cost was used as this model is less sensitive to outliers compared to ordinary least squares regression [29]. In this analysis, all variables considered to potentially predict the cost incurred by patients and/or their families were included in both unadjusted and adjusted models employed. In the assessment of the independent variables to be included in the models, variance inflation factor (VIF) was determined and the standardized generalized VIF values of all the independent variables ranged from 1.09 to 1.52. This indicated that it was within the acceptable limit to proceed with all the variables in the model. The quantile regression part of the analysis was done using the software, R version 3.4.4 [30]. In grouping the participants into the five wealth quintiles, principal component analysis was employed. The categorization of households of patients in the study into wealth quintiles was on the basis of their answers to questions regarding the availability of a range of household properties (e.g. household items, land, farm animals, vehicle among others). In all the analyses, statistical significance of possible associations were determined using *p*-value< 0.05 as a cut-off at 95% CI.

## Results

### Socio-demographic profile

In this study, interviews with 342 patients, out of a total of 346 encounters, were included in the final analysis. The four interviews were excluded due to incompleteness making a response rate of 98.8%. Table 1 shows the socio-demographic distribution of the participants in the study. Patients in the age group of 18–29 years constituted the highest proportion (41.2%), while women made up nearly two-thirds (61.1%) of the participants. In terms of educational status, those unable to read and write constituted a leading proportion, accounting for more than a third of the participants (33.9%). More than one-third (36.3%) of the participants were housewives followed by farmers (22.2%).

**Table 1:** Socio-demographic profile of the outpatients (*N* = 342)

Variable	Frequency (%)
Age (years)	
18–29	141 (41.2)
30–39	84 (24.6)
40–49	46 (13.5)
50–59	45 (13.2)
60+	26 (7.6)
Sex	
Male	133 (38.9)
Female	209 (61.1)
Marital status	
Married	195 (57.0)
Unmarried	100 (29.2)
Divorced/separated	27 (7.9)
Widow/er	20 (5.8)
Educational status	
Unable to read and write	116 (33.9)
Able to read and write	33 (9.6)
Primary school (Grades 1–8)	72 (21.1)
Secondary school (9–10)	55 (16.1)
College preparatory level	14 (4.1)
Technical and vocational education and training	31 (9.1)
University education	21 (6.1)
Major occupation	
Government employee	36 (10.5)
Private company employee	18 (5.3)
Self-employed/business person	16 (4.7)
Housewife	120 (35.1)
Farmer	76 (22.2)
Student	43 (12.6)
Unemployed	21 (6.1)
Other ^a^	12 (3.5)
Permanent residence	
Gondar town	120 (35.1)
Areas outside Gondar town	222 (64.9)
Family size (in number)	
One to two	110 (32.2)
Three to four	101 (29.5)
Five to seven	85 (24.9)
Eight or more	46 (13.5)
Number of working family members	
One	160 (46.8)
Two	115 (33.6)
Three or more	67 (19.6)
Wealth index of family	
Lowest	68 (19.9)
Lower	69 (20.2)
Middle	68 (19.9)
Higher	69 (20.2)
Highest	68 (19.9)

^a^Daily laborer, driver

Table 1 also shows the family aspect of the participants’ socio-demographic information. The majority of participants, accounting for nearly two-thirds (64.9%), were from areas outside Gondar town; while about a third (32.2%) came from families with one to two members, followed by those from families with three to four members (29.5%). Among the participants, nearly half of the (46.8%) reported having one member earning income for the family. The economic status of the families of the participants was presented divided into wealth quintiles based on ownership of a variety of household items.

### Features of patients’ visit to the hospital

In Table 2, the features of visit the participants made to the hospital are summarized. The majority of visits made to the hospital by the participants were for follow-up on previous visits mainly chronic illnesses, accounting for nearly two-thirds (65.5%). More than half of the participants (54.7%) came to the hospital with a caregiver. As to payment scheme, more than three quarters (81.0%) of the patients reported that the expenses, related to the health care services they received at the hospital, were covered by themselves or their families. Table 3, shows the most common illnesses reported by patients as reasons for visit to the hospital.

**Table 2:** Features of patients’ visit to the hospital (*N* = 342)

Variable	Frequency (%)
Reason for visit	
Newly occurred illness	118 (34.5)
Follow-up for chronic illness	224 (65.5)
Accompanied by caregiver to hospital	
Yes	187 (54.7)
No	155 (45.3)
Payment scheme	
Patient/family	277 (81.0)
Free of fee charge	62 (18.1)
Others^a^	3 (0.9)

^a^Paid by employers, paid partly by employers

**Table 3:** The five most common illnesses reported by patients as reasons for the hospital

Reported illness	Frequency	Percentage
Gastrointestinal related illnesses	53	15.5
Mental ill health	36	10.5
Diabetes	23	6.7
Skin related illnesses	22	6.1
Heart failure	17	5.0

### Costs associated with action taken before visit to the hospital

**Fig. 1:**
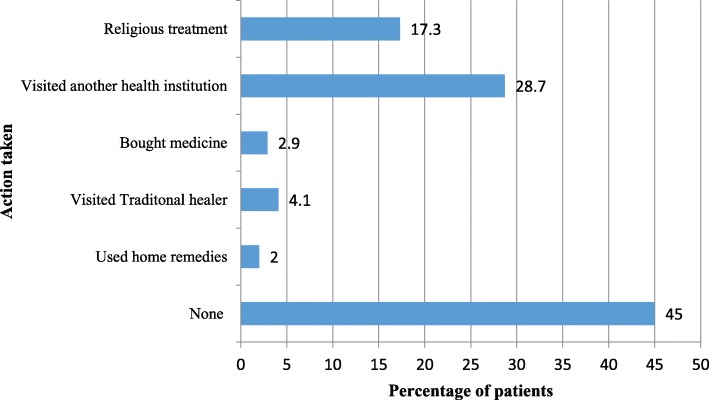
Proportions of the types of actions taken before a visit to the hospital (*N* = 342)

Among the participants in the study, more than half (55.0%) reported they took some form of treatment before their visit to the hospital. Figure 1 illustrates the different actions taken, with a visit to another health institution made by more than a quarter of the participants (28.7%). The mean and median OOP costs incurred before hospital visit were 10.87 USD (standard deviation (SD) =45.14) and zero USD (inter-quartile range (IQR) = 0.00–2.06 USD).

### Direct medical costs at the hospital

Table 4 shows the cost components of direct medical costs incurred by participants and their families. The median direct medical cost was calculated to be 1.83 USD (IQR = 0.23–5.36 USD). Medicines (median = 0.62 USD, IQR = 0.00–1.74) and consultation fees (median = 0.23 USD, IQR = 0.00–0.23) were major contributors of direct medical cost.

**Table 4:** Direct medical cost incurred by participants for hospital visit (*N* = 342)

Cost type	Median	IQR
Physician consultation	0.23	0.00–0.23
Diagnostic /laboratory	0.00	0.00–2.67
Medicine(s)	0.62	0.00–1.74
Injection and/or other services	0.00	0.00–0.00
Total (USD)	1.83	0.23–5.36

### Direct nonmedical costs

OOP spending on the nonmedical component of the hospital visits made by participants of the study included transportation, food and accommodation expenses. Table 5 presents these cost costs, both for patients and caregivers accompanying them to the hospital. Median total direct nonmedical cost spent for patients and caregivers were 2.75 USD (IQR = 0.46–8.24 USD) and 0.27 USD (IQR = 0.00–3.89 USD) respectively. In both cases, transportation cost was the major component.

**Table 5:** Direct nonmedical cost spent by patients and caregivers (*N* = 342)

Individuals	Cost component	Median	IQR
Patient-related	Transportation	1.83	0.37–3.67
	Food	0.46	0.00–2.29
	Accommodation	0.00	0.00–0.00
	Total	2.75	0.46–8.24
Care-giver related	Transportation	0.21	0.21–0.75
	Food	0.00	0.00–0.69
	Accommodation	0.00	0.00–0.00
	Total	0.27	0.00–3.89
Total direct nonmedical cost (USD)		4.58	0.73–13.05

### Indirect cost

In Table 6, the components of indirect cost are summarized, which included loss before, during travel to and from hospital and stay during hospital visit. The median total time lost was found to be more than 4 days. The time loss associated to stay during services at the hospital was the main contributor to time loss related to both patients and their caregivers.

**Table 6:** Patient and caregiver related time due to illness/condition (in number of days) (*N* = 342)

Individuals	Time lost (days)	Median	IQR
Patient related	Before visit to hospital	0.00	0.00–3.00
	During travel	0.06	0.02–0.12
	During stay for hospital care	2.00	0.21–3.00
	Total	2.38	1.01–7.03
Caregiver related	Before visit to hospital	0.00	0.00–1.25
	During travel	0.02	0.00–0.08
	During stay for hospital care	0.09	0.00–2.00
	Total	0.82	0.00–4.12
Total time loss		4.08	1.45–14.09

Looking at the monetary value of the time lost, a median total of 3.66 USD (IQR = 0.00–21.99 USD) was lost due to the outpatient visit. Specific costs of time loss related to patients and caregivers, had median costs of zero USD with IQRs of 0.00 to 8.08 USD and 0.00 to 5.85 USD respectively.

### Total cost incurred by outpatients and their families

As described in Table 7, the median total cost borne by outpatients and their families who visited the hospital was 22.25 USD (IQR = 7.17–56.99 USD). The main drivers of the total cost were direct nonmedical and indirect costs such as transportation, and lost time due to stay for the hospital visit respectively.

**Table 7:** Components of total cost incurred by patients (in USD) (*N* = 342)

Cost component	Median	IQR
Direct cost	10.76	3.42–27.41
Indirect cost	3.66	0.00–21.09
Total cost	22.25	7.17–56.99

### Association of cost with socio-demographic variables

On the basis of the findings from the quantile regression (median regression), in the unadjusted regression models, sex, age group, educational status, occupational status and wealth index were found to be associated with median total cost. However, in the adjusted model, these variables did not retain their statistically significant associations. The variables residence, marital status and payment scheme were found to predict median total cost incurred by patients and their families after controlling for other socio-demographic variables (Table 7).

Looking specifically at residence, patients living outside the town of Gondar were found to incur more than ten times the median cost of those who came to the hospital from within the town, after controlling for other variables. In the adjusted model, unmarried patients were also found to have incurred about ten times higher median total cost compared to married patients. In addition, patients served free of charge in the hospital incurred nearly 15 times less median total cost compared to those who covered the payments at the hospital by themselves or through their families (Table 8).

**Table 8:** Quantile regression (median) of total cost incurred by socio-demographic factors

Variable	Unadjusted coefficient	95% CI	Adjusted coefficient	95% CI
Sex^c^				
Female	−7.156	[−15.790, −2.050]^*^	−2.961	[−9.460, 2.132]
Residence^d^				
Outside Gondar	20.935	[16.480, 27.023]^*^	10.948	[6.896, 25.362]^*^
Reason for visit^e^				
Follow up visit	5.969	[−0.412, 11.926]	4.456	[−3.313, 8.999]
Age(years)^f^				
30–39	3.916	[−1.957, 11.494]	6.327	[−4.358, 12.242]
40–49	9.412	[2.488, 19.437]^*^	7.319	[−2.937, 18.748]
50–59	7.208	[−1.062, 18.262]	4.446	[−6.598 17.703]
60+	14.301	[6.103, 34.680]^*^	15.852	[−1.362, 34.684]
Marital status^g^				
Unmarried	−3.970	[−9.600, 2.988]	10.250	[0.923, 20.903]^*^
Divorced/separated	−8.154	[−14.596, − 1.281]	−6.367	[− 12.448 14.329]
Widow/er	− 12.214	[−24.808, 9.439]	−2.822	[− 12.767, 11.505]
Educational status^h^				
Able to read and write	−5.770	[− 13.779, 4.816]	− 6.641	[− 21.885, 11.028]
Primary school (Grades 1–8)	− 10.900	[− 20.019, − 6.340]^*^	−7.930	[−15.613, 2.467]
Secondary school (Grades 9–10)	−10.165	[− 18.504, − 3.234]^*^	−8.063	[− 15.390, 0.993]
University education	− 13.955	[− 27.328, − 3.363]^*^	− 2.953	[− 20.775, 9.309]
Major occupation^i^				
Private company employee	8.579	[− 3.229, 55.444]	0.096	[− 20.537, 45.658]
Self-employed/ business person	−3.401	[−18.657, 7.019]	− 12.006	[− 30.692, 1.768]
Housewife	− 1.147	[− 15.782, 3.234]	−16.096	[− 32.824, −0.954]
Farmer	9.238	[− 3.533, 18.309]	−9.862	[− 31.740, 8.266]
Student	−9.230	[− 20.167, −0.698]^*^	− 24.374	[−43.469, − 4.505]
Other ^a^	−18.253	[− 29.113, − 5.950]^*^	−17.159	[− 33.342, − 3.041]
Family size (number)	0.356	[− 0.556, 2.623]	0.033	[− 1.207, 1.399]
Number of working family members	3.936	[− 0.072, 9.983]	2.801	[− 0.912, 5.966]
Wealth index of family^j^				
Lower	8.720	[−3.045,16.050]	−2.475	[− 6.864, 5.230]
Middle	13.936	[7.131,17.868]^*^	−6.163	[− 14.672, 7.732]
Higher	17.942	[12.424,24.872]^*^	−1.585	[−16.565, 12.629]
Highest	19.196	[11.616,28.365]^*^	−0.314	[−10.427, 11.621]
Payment^k^				
Free of fee	−19.201	[−22.750, − 13.416]^*^	− 14.907	[−20.892, −7.799]^*^
Other^b^	−6.754	[− 13.321, − 6.517]^*^	−11.181	[− 19.827, 14.231]

Reference: ^c^Male, ^d^Gondar, ^e^new, ^f^18–29, ^g^married, ^h^unable to read and write, ^i^government employee, ^j^lowest, ^k^patient/family

^*^P-value < 0.05 ^a^ Daily labourer, driver ^b^ Paid by employers, paid partly by employers

## Discussion

The study assessed costs incurred by patients and/or their families due to illnesses/conditions which required outpatient visits to the hospital. The median total cost incurred by patients was found to be more than 22.25 USD per visit. Of this, median direct and indirect costs were found to be 10.76 USD and 3.66 USD respectively. Residence, marital status and payment scheme were found to predict median total cost controlling for confounders in the adjusted model.

The patients incurred a significantly high median total cost of more than 22 USD per visit. Of this, direct nonmedical and indirect costs were the main components. This shows that patients and/or their families incur hidden costs to cover for their travel to the hospital and the loss of time they experience in the process beside the obvious expense for medical care at the hospital. A comparable mean total cost of outpatient visit, 23.7 USD (2012), was reported by a study from Lao People’s Democratic Republic (PDR) [31]. Another study from Bangladesh, which focused on cost of patient visits to public (132.31 USD) and private hospitals (74.77 USD), reported higher mean costs (June 2011 value) [32].

Looking at direct costs, a median of 10.76 USD was incurred by patients, which was comparable to a finding from Bolivia. The latter study reported on a median OOP cost incurred by caregivers of children with diarrhea from urban (10.74 USD) and rural areas (17.63 USD) [33]. The finding in the present study was similar to that from southern Ethiopia, where households paid a median of 10.73 USD for antenatal care in public health facilities [19]. Another comparable finding was also reported by a study from Albania among acutely ill patients [34]. As to indirect costs, the present study reported a lower median cost compared to the finding from Lao PDR (6.3 USD, 2012) [31]. The study from Bolivia reported lower indirect cost for urban and higher for rural areas [33].

Looking at cost drivers, the major contribution towards the direct cost in the present study was by nonmedical expenses with a lower contribution from medical cost. However, unlike the result of this study, different studies reported that direct medical cost accounted for a major share of the total OOP payment. These included the findings from Albania and India where direct medical costs contributed high proportions of the total OOP [34, 35]. Similarly, in the study from Lao PDR, 65% of the OOP for the outpatient visit was contributed by direct medical costs [31]. The study on costs associated with pneumonia (6 versus 2 USD) and diarrhea (5 versus 2 USD) in Ethiopia, reported a similarly higher proportion of direct medical costs compared to direct nonmedical costs [22]. The difference from the present study could be attributed to the high proportion of patients who came from outside Gondar town, which could have contributed to higher cost associated with travel and other amenities. The fee waiver for the poor and exemptions for selected services such as maternal care, could also explain the difference [36].

The major component of indirect cost was found to be the wait at the hospital, for both patient and caregiver related time loss. Long waiting time has also been cited as one of the important components of indirect cost in another study [32]. The waiting times associated to hospital services are commonly overlooked, however, they have a significant cost to patients/families as demonstrated here. The shortage of health professionals and infrastructural issues in the hospital studied might have contributed to longer waits for service in the hospital.

The median total cost was found to differ with a number of socio-demographic variables. Of these, median total cost incurred by patients/families was found to differ by permanent residence, where patients from outside Gondar incurred much higher cost compared to those from within the town. The high direct nonmedical and indirect costs, major drivers of the median total cost in the outpatient visit, might have contributed to the difference. Similarly higher median total cost was reported for patients form rural areas compared to those from urban areas by a study from Bolivia [33]. Despite improvements made in health services in rural parts of Ethiopia, a lot remains to be done both in access and quality of health services [37].

Marital status showed different associations with median total cost in the unadjusted and adjusted regression models. Although it did not show statistically significant predictive effect in the unadjusted regression model, being unmarried was found to be associated with higher median total cost in the adjusted model where other socio-demographic variables were controlled for. One possible explanation could be the statistically significant variation in the proportion of men and women in different marital status groups, with higher proportion of men in the unmarried group unlike the rest where women constituted higher proportions. As men incurred higher cost, although not statistically significant, compared to women, the higher median cost among unmarried patients could be related to higher proportion of men in the group.

In regard to payment for services at the hospital, patients who were provided with waiver/ served free of fee incurred lower total median cost compared to those who paid by themselves, which showed the waiver provided by the hospital affected the overall expense incurred by patients. At the hospital patients who were verified to not be able to afford to pay and women receiving maternal services are provided services free of charge. Hence, the significant proportion of such patients contributed to the much lower median total cost among such patients and/or their families.

The findings from the present study are based on one university referral hospital. However, it has similar referral and patronage by patients like other hospitals in the region where the hospital is located as well as in other parts of the country. Although it may not be wholly generalizable to other hospitals in Ethiopia, it provides insight into the amount and components of cost incurred by patients in Ethiopia.

### Limitations

The study has a limitation as to the fact that it involved interviewing of patients who came to the outpatient pharmacy of the hospital after they received services in all other parts of the hospital. So, patients who left the hospital with no medicine prescribed to them were not represented in this finding. As the data was collected in a limited period of time, possible seasonal variations which may affect the level of cost incurred by patients may not be fully captured by the current findings.

## Conclusions

Outpatients visiting GURH incurred a significant amount of cost with direct nonmedical and indirect costs being major components. The cost of medicines from direct medical cost, transportation from direct nonmedical, and time lost due to hospital stay took major share. The total median cost was different by residence, marital status, as well as payment scheme.

As an effort to bring down the cost faced by outpatients, strengthening of primary health care institutions closer to patients’ residence is recommended. The improvement of such services can be helpful in helping reduce time lost due to visits and cost related to travel and other services. In addition, further studies with samples more representative of patient visits throughout the year and more detail look into specific diagnoses needs to be done to help analyze the cases associated with the hospital visit and their cost implications.

## Abbreviations

CI: Confidence interval
DRC: Democratic Republic of the Congo
ETB: Ethiopian Birr
HIV/AIDS: Human Immunodeficiency Virus
IQR: Inter-quartile range
Lao PDR: Lao People’s Democratic Republic
OOP: Out-of-pocket
SD: Standard deviation
THE: Total health expenditure
USD: United States Dollars
WHO: World Health Organization
